# Supplementary material for: The Evolution and Characterization of the RNA Interference Pathways in Lophotrochozoa
Source: Genome Biol Evol. 2024 May 7;16(5):evae098. doi: 10.1093/gbe/evae098 (PMC11114477; doi:10.1093/gbe/evae098)
Supplement: evae098_Supplementary_Data [file evae098_supplementary_data.zip › Supplementary_materials.zip!/selected_miRNAs/NoGe_selected_miRNAs/mirdeep_pdf_files/NMRB01003515.1_Notospermus_geniculatus_strain_Ushimado_scaffold3515,_whole_genome_shotgun_sequence_43826.pdf]

Provisional ID : NMRB01003515.1_Notospermus_geniculatus_strain_Ushimado_scaffold3515,_whole_genome_shotgun_sequence_43826
Score total : 37806.6
Score for star read(s) : 3.9
Score for read counts : 37798.1
Score for mfe : 1.6
Score for randfold : 0
Score for cons. seed : 3
Total read count : 74151
Mature read count : 74142
Loop read count : 0
Star read count : 9

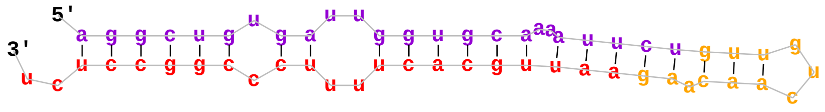

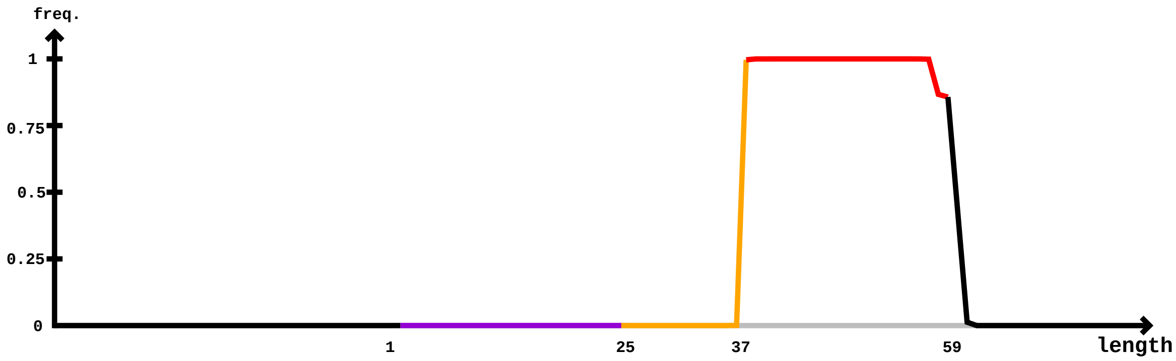


```
                                          Star                                       Mature
5'- accccaaacccuggaucucccggcugcuuagcaaaggcugugauuggugcaaaauucuguugucaacaagaauugcacuuuucccggccucugugcuguggccaguucuucc  -3'  obs
    accccaaacccuggaucucccggcugcuuagcaaaggcugugauuggugcaaaauucuguugucaacaagaauugcacuuuucccggccucugugcuguggccaguucuucc       exp
    ...(((.....))).......((((((...(((.((((((.((..((((((..((((((((...))).)))))))))))..)).)))))).)))...))))))......... reads  mm  sample
    ......................................................................aauugcacuuuucccggc........................ 1      0   Fm3
    ......................................................................aauugcacuCuucccggcc....................... 1      1   Fm3
    ......................................................................aauugcacuuAucccggccu...................... 1      1   Fm3
    ......................................................................aauugcacuuuucccggccu...................... 136    0   Fm3
    ......................................................................aauugcacuuuucccggAcu...................... 1      1   Fm3
    ......................................................................aauugcacuuuucccggGcu...................... 2      1   Fm3
    ......................................................................Uauugcacuuuucccggccu...................... 1      1   Fm3
    ......................................................................aauugcacuuCucccggccu...................... 1      1   Fm3
    ......................................................................aauugcacuuuucccggccuG..................... 1      1   Fm3
    ......................................................................aauugcacuuuucccggccuGu.................... 11     1   Fm3
    ......................................................................Uauugcacuuuucccggccucu.................... 1      1   Fm3
    ......................................................................aauugcacuuuucccggccucA.................... 1      1   Fm3
    ......................................................................aauugcacuuuucccggccucu.................... 20     0   Fm3
    ......................................................................aauugcacuuGucccggccucu.................... 1      1   Fm3
    .......................................................................auugcacuuuucccggccuGu.................... 4      1   Fm3

    ..................................aggcugugauuggugcaaaauucu...................................................... 1      0   Mm3
    .....................................................................Caauugcacuuuucccggccu...................... 1      1   Mm3
    .....................................................................gaauugcacuuuucccggccu...................... 1      0   Mm3
    .....................................................................Caauugcacuuuucccggccucu.................... 5      1   Mm3
    ......................................................................aauugcacuuuucccggc........................ 4      0   Mm3
    ......................................................................aauugcacuuuucccggU........................ 1      1   Mm3
    ......................................................................aauugcacuCuucccggcc....................... 1      1   Mm3
    ......................................................................aauugcacuuuucccggcc....................... 34     0   Mm3
    ......................................................................aauugcacuuuucccggcA....................... 1      1   Mm3
    ......................................................................aauugcacuuuucccggcU....................... 2      1   Mm3
    ......................................................................aauugcacuuuuccUggccu...................... 1      1   Mm3
    ......................................................................aauugcacuuuucccAgccu...................... 1      1   Mm3
    ......................................................................aauugcacuuuucccgCccu...................... 5      1   Mm3
    ......................................................................Gauugcacuuuucccggccu...................... 1      1   Mm3
    ......................................................................aauugcacuuuucccggccA...................... 17     1   Mm3
    ......................................................................aauugcacuuuucccgAccu...................... 4      1   Mm3
    ......................................................................aauugcacuuuucccggUcu...................... 16     1   Mm3
```

Star **Mature**

```
accccaaacccuggaucucccggcugcuuagcaaaggcugugauuggugcaaaauucuguugucaacaagaauugcacuuuucccggccucugugcuguggccaguucuucc
......................................................................aauugcacuuuucccgUccu......................	5	1	Mm3
......................................................................Nauugcacuuuucccggccu......................	3	1	Mm3
......................................................................aauuCcacuuuucccggccu......................	2	1	Mm3
......................................................................aauugcacuuuucccggccu......................	2182	0	Mm3
......................................................................aauugNacuuuucccggccu......................	1	1	Mm3
......................................................................Uauugcacuuuucccggccu......................	3	1	Mm3
......................................................................aauuAcacuuuucccggccu......................	1	1	Mm3
......................................................................aauugcacuCuucccggccu......................	5	1	Mm3
......................................................................aauugcacuuuucccggGcu......................	6	1	Mm3
......................................................................aaGugcacuuuucccggccu......................	1	1	Mm3
......................................................................aauugcacuuuucccggccC......................	8	1	Mm3
......................................................................aauugcacuuuuGccggccu......................	1	1	Mm3
......................................................................aauugcacuuCucccggccu......................	2	1	Mm3
......................................................................aauugcacuuuuUccggccu......................	1	1	Mm3
......................................................................aUuugcacuuuucccggccu......................	1	1	Mm3
......................................................................aaCugcacuuuucccggccu......................	1	1	Mm3
......................................................................aauCgcacuuuucccggccu......................	1	1	Mm3
......................................................................aauugcacuuuucccggccG......................	46	1	Mm3
......................................................................aauugcacuuuucccggAcu......................	23	1	Mm3
......................................................................aauugcacuuAucccggccu......................	8	1	Mm3
......................................................................aauugcacuuuucccggccCc.....................	2	1	Mm3
......................................................................aauugcacuuuucccggAcuc.....................	2	1	Mm3
......................................................................aauugcacuuuucccCgccuc.....................	1	1	Mm3
......................................................................aauugcacuuuucccggccuc.....................	127	0	Mm3
......................................................................aauugcacuuuucccggccuU.....................	39	1	Mm3
......................................................................aauugcacuuuucccggccuG.....................	7	1	Mm3
......................................................................aauugcacuuAucccggccuc.....................	1	1	Mm3
......................................................................aauugcacuuGucccggccuc.....................	1	1	Mm3
......................................................................aauugcacuuuuccAggccucu....................	1	1	Mm3
......................................................................aauugcacuuAucccggccucu....................	11	1	Mm3
......................................................................aauugcacuuuucccggccCcu....................	1	1	Mm3
......................................................................Nauugcacuuuucccggccucu....................	7	1	Mm3
......................................................................aauugcacuuuucccggccucu....................	9576	0	Mm3
......................................................................aauugcacuuuucccggAcucu....................	77	1	Mm3
......................................................................aauugcacuuuuccUggccucu....................	3	1	Mm3
......................................................................aauugcacuuuucccggccucC....................	7	1	Mm3
......................................................................aauugcacuuuucccggUcucu....................	70	1	Mm3
......................................................................aGuugcacuuuucccggccucu....................	1	1	Mm3
......................................................................aauugcacuuuucAcggccucu....................	5	1	Mm3
......................................................................aauAgcacuuuucccggccucu....................	1	1	Mm3
......................................................................aauugcGcuuuucccggccucu....................	2	1	Mm3
......................................................................aauugcacuuGucccggccucu....................	42	1	Mm3
......................................................................aauugcacuuuucccUgccucu....................	2	1	Mm3
......................................................................aauuAcacuuuucccggccucu....................	2	1	Mm3
......................................................................aauugcacuuCucccggccucu....................	13	1	Mm3
......................................................................aaCugcacuuuucccggccucu....................	2	1	Mm3
......................................................................aauugcacuuuucccggccuAu....................	3	1	Mm3
......................................................................aauugcaUuuuucccggccucu....................	1	1	Mm3
......................................................................aauugcacuuuucccggccucG....................	90	1	Mm3
......................................................................aauugcacuGuucccggccucu....................	1	1	Mm3
......................................................................aCuugcacuuuucccggccucu....................	1	1	Mm3
......................................................................aauugcacuuuucccgAccucu....................	24	1	Mm3
......................................................................aaAugcacuuuucccggccucu....................	1	1	Mm3
......................................................................aauugcacuuuucccgCccucu....................	19	1	Mm3
......................................................................aauugcacuuuuUcggccucu....................	3	1	Mm3
......................................................................aauCgcacuuuucccggccucu....................	2	1	Mm3
......................................................................aUuugcacuuuucccggccucu....................	1	1	Mm3
......................................................................Uauugcacuuuucccggccucu....................	11	1	Mm3
......................................................................aauugcacuuuucccgUccucu....................	17	1	Mm3
......................................................................aauugcacuuuucccggccucA....................	13	1	Mm3
......................................................................aauugcacuuuAcccggccucu....................	2	1	Mm3
......................................................................aauugcacuuuCcccggccucu....................	3	1	Mm3
......................................................................aauugcacuuuuccAgccucu....................	3	1	Mm3
......................................................................aauugcacuuuuUccggccucu....................	1	1	Mm3
......................................................................aauuUcacuuuucccggccucu....................	3	1	Mm3
......................................................................aauugcacuuuucccggGcucu....................	30	1	Mm3
......................................................................aauugcUcuuuucccggccucu....................	2	1	Mm3
......................................................................aauugAacuuuucccggccucu....................	2	1	Mm3
......................................................................aauugcacuuuucccggccuGu....................	115	1	Mm3
......................................................................aauGgcacuuuucccggccucu....................	1	1	Mm3
```

```
                                  Star                              Mature
accccaaacccuggaucucccggcugcuuagcaaaggcugugauuggugcaaaauucuguugucaacaagaauugcacuuuucccggccucugugcuguggccaguucuucc
......................................................................Gauugcacuuuucccggccucu....................    1     1    Mm3
......................................................................aauugUacuuuucccggccucu....................    4     1    Mm3
......................................................................aauugcCcuuuucccggccucu....................    1     1    Mm3
......................................................................aauugcacuuuucccggccuUu....................    5     1    Mm3
......................................................................aauugcacuuuucccggccucuC...................    1     1    Mm3
......................................................................aauugcacuuuucccggccucuA...................    52    1    Mm3
......................................................................aauugcacuuuucccggccucuU...................    451   1    Mm3
......................................................................aauugcacuuuucccggccucuUu..................    102   1    Mm3
......................................................................aauugcacuuuucccggccucuAu..................    41    1    Mm3
.......................................................................auugcacuuuucccggccuc.....................    1     0    Mm3
.......................................................................auugcacuuuucccggccuGu....................    3     1    Mm3
.......................................................................auugcacuuuucccggcAucu....................    1     1    Mm3
.......................................................................auugcacuuuGcccggccucu....................    1     1    Mm3
.......................................................................auugcacuuuuUccggccucu....................    1     1    Mm3
.......................................................................auugcacuuuucccggccucu....................    27    0    Mm3
.......................................................................auugcacuuuucccggccucuA...................    2     1    Mm3
.......................................................................auugcacuuuucccggccucuU...................    5     1    Mm3
.......................................................................auugcacuuuucccggccucuC...................    1     1    Mm3
.......................................................................auugcacuuuucccggccucug...................    1     0    Mm3
.......................................................................auugcacuuuucccggccucugA..................    1     1    Mm3
.......................................................................auugcacuuuucccggccucuAu..................    3     1    Mm3
.......................................................................auugcacuuuucccggccucugu..................    5     0    Mm3

......................................................................aauugcacuuuucccgAccu......................    3     1    Fm2
......................................................................aauugcacuuuucccggAcu......................    13    1    Fm2
......................................................................aauuUcacuuuucccggccu......................    1     1    Fm2
......................................................................aauugcacuuuucccgCccu......................    1     1    Fm2
......................................................................aauugcacuuuucccggccu......................    764   0    Fm2
......................................................................aUuugcacuuuucccggccu......................    1     1    Fm2
......................................................................aauugcacuuAucccggccu......................    1     1    Fm2
......................................................................aauugcacuuuucccggUcu......................    3     1    Fm2
......................................................................Gauugcacuuuucccggccu......................    1     1    Fm2
......................................................................aauugcacuuCucccggccu......................    1     1    Fm2
......................................................................aauugcacuuuucccggccG......................    39    1    Fm2
......................................................................aauugcacuuuucccgUccu......................    1     1    Fm2
......................................................................aaGugcacuuuucccggccu......................    1     1    Fm2
......................................................................aauugcacGuuucccggccu......................    1     1    Fm2
......................................................................aauugcacuuuucccggcGu......................    1     1    Fm2
......................................................................Uauugcacuuuucccggccu......................    1     1    Fm2
......................................................................aauugcacuuuucccggGcu......................    2     1    Fm2
......................................................................aauugcacuuuucUcggccu......................    1     1    Fm2
......................................................................aauugcacuuuuccUggccu......................    2     1    Fm2
......................................................................aauugcacuuuucccggccuU.....................    18    1    Fm2
......................................................................aauugcacuuuucccggccuc.....................    54    0    Fm2
......................................................................aauugcacuuuucccggAcuc.....................    1     1    Fm2
......................................................................aauuUcacuuuucccggccucu....................    1     1    Fm2
......................................................................aauugcacuuuucAcggccucu....................    6     1    Fm2
......................................................................aaCugcacuuuucccggccucu....................    1     1    Fm2
......................................................................aauugcacuuuucccggccucu....................    5418  0    Fm2
......................................................................aauugcacuuuuccGggccucu....................    1     1    Fm2
......................................................................aauugcacuuuucccggccCcu....................    1     1    Fm2
......................................................................aauugcacuuCucccggccucu....................    6     1    Fm2
......................................................................Gauugcacuuuucccggccucu....................    2     1    Fm2
......................................................................aauAgcacuuuucccggccucu....................    1     1    Fm2
......................................................................aauugcacuuuucccggcAucu....................    1     1    Fm2
......................................................................aauugcacCuuucccggccucu....................    4     1    Fm2
......................................................................Nauugcacuuuucccggccucu....................    1     1    Fm2
......................................................................aCuugcacuuuucccggccucu....................    2     1    Fm2
......................................................................aauugcacuuuucccggccucA....................    2     1    Fm2
......................................................................aauugcacuuuucccggccuUu....................    2     1    Fm2
......................................................................aauugcacuuAucccggccucu....................    7     1    Fm2
......................................................................aauugcacuuuucccggUcucu....................    33    1    Fm2
......................................................................aauuCcacuuuucccggccucu....................    1     1    Fm2
......................................................................aauugcacuuuucccggcUucu....................    1     1    Fm2
......................................................................aauugcacuuuuccAgccucu....................    2     1    Fm2
......................................................................aauugcacuuuucccggccucC....................    8     1    Fm2
......................................................................aauugcacuuuuUcggccucu....................    5     1    Fm2
......................................................................aauugcaUuuuucccggccucu....................    1     1    Fm2
......................................................................aauugcacuuuuUccggccucu....................    1     1    Fm2
......................................................................aauugGacuuuucccggccucu....................    2     1    Fm2
```

Star Mature

accccaaacccuggaucucccggcugcuuagcaaaggcugugauuggugcaaaauucuguugucaacaagaauugcacuuuucccggccucugugcuguggccaguucuucc			
....aauugcacuuuucccgUccucu....................	13	1	Fm2
....Uauugcacuuuucccggccucu....................	10	1	Fm2
....aauugcacuuGucccggccucu....................	3	1	Fm2
....aauugcGcuuuucccggccucu....................	2	1	Fm2
....aauugcacuuuucccggccucG....................	111	1	Fm2
....Cauugcacuuuucccggccucu....................	2	1	Fm2
....aauugcacuuuuccUggccucu....................	2	1	Fm2
....aauugcacAuuucccggccucu....................	1	1	Fm2
....aauugcacuuuucccggGcucu....................	21	1	Fm2
....aauugcacuuuucccgAccucu....................	11	1	Fm2
....aauuAcacuuuucccggccucu....................	1	1	Fm2
....aauugUacuuuucccggccucu....................	1	1	Fm2
....aauugcacuuuucccggAcucu....................	46	1	Fm2
....aauugcacuuuucccgCccucu....................	14	1	Fm2
....aauugcacuuuuccAggccucu....................	2	1	Fm2
....aauugcacuuuucccggccucuU...................	279	1	Fm2
....aauugcacuuuucccggccucug...................	3	0	Fm2
....aauugcacuuuucccggccucuA...................	76	1	Fm2
....aauugcacuuuucccggccucuAu..................	30	1	Fm2
....aauugcacuuuucccggccucuUu..................	116	1	Fm2
....auugcacuuuucccggccucu....................	5	0	Fm2
....auugcacuuuucccggccucuA...................	2	1	Fm2
................................aaaggcugugauuggugcaaaauu..	3	0	Fm1
..................................aggcugugauuggugcaaaauucu..	3	0	Fm1
....Caauugcacuuuucccggccucu....................	2	1	Fm1
....aauugcacuCuucccggc........................	1	1	Fm1
....aauugcacuuuucccggcc.......................	4	0	Fm1
....aauugcacuuuucccgAccu......................	3	1	Fm1
....aauugcacuuuucccggccC......................	1	1	Fm1
....aauugcacuuuucUcggccu......................	2	1	Fm1
....aauugcCcuuuucccggccu......................	1	1	Fm1
....aauugcacuuAucccggccu......................	6	1	Fm1
....Uauugcacuuuucccggccu......................	1	1	Fm1
....aauugcacuuuucccggccu......................	2117	0	Fm1
....aauugcacuuuucccgUccu......................	3	1	Fm1
....aauugcacCuuucccggccu......................	1	1	Fm1
....aauugcacuuuucccggAcu......................	23	1	Fm1
....aauugNacuuuucccggccu......................	1	1	Fm1
....aauugcacuuuucccggccG......................	92	1	Fm1
....aauugcacuuuucccggGcu......................	11	1	Fm1
....aCuugcacuuuucccggccu......................	1	1	Fm1
....aauugcacuCuucccggccu......................	1	1	Fm1
....aauugcacuuuucccggccA......................	6	1	Fm1
....aauugcacuuuucccggUcu......................	5	1	Fm1
....aauugcacuuuucccgCccu......................	7	1	Fm1
....aauugcacuuuuUccggccuc.....................	1	1	Fm1
....aauugcacuuuucccggccuU.....................	19	1	Fm1
....aauugcacuuuucccggccuc.....................	114	0	Fm1
....aauugcacuuuucccgCccuc.....................	1	1	Fm1
....Uauugcacuuuucccggccuc.....................	1	1	Fm1
....aauugcacuuuucccggccuA.....................	5	1	Fm1
....aauugcacuuuucccAgccuc.....................	1	1	Fm1
....aauugcacuuuucccgUccuc.....................	1	1	Fm1
....aauCgcacuuuucccggccucu....................	3	1	Fm1
....aauugcacuuuuUccggccucu....................	19	1	Fm1
....aauugcacAuuucccggccucu....................	1	1	Fm1
....aauuCcacuuuucccggccucu....................	1	1	Fm1
....aauugcacuuuucccggccuGu....................	7	1	Fm1
....aauugcacuuuucccggGcucu....................	29	1	Fm1
....aauugcacuuuucccgCccucu....................	16	1	Fm1
....aauugcacuuuucccggUcucu....................	52	1	Fm1
....aauugcacuuuucccggccuUu....................	14	1	Fm1
....aauugcacCuuucccggccucu....................	8	1	Fm1
....aaGugcacuuuucccggccucu....................	2	1	Fm1
....aauugcacuGuucccggccucu....................	3	1	Fm1
....aauugcacuuuucccggAcucu....................	93	1	Fm1
....aauugcacuuuucccggccGcu....................	1	1	Fm1
....aauugcacuuuucccggccucu....................	8214	0	Fm1
....aCuugcacuuuucccggccucu....................	3	1	Fm1

```
                                  Star                                   Mature
accccaaacccuggaucucccggcugcuuagcaaaggcugugauuggugcaaaauucuguugucaacaagaauugcacuuuucccggccucugugcuguggccaguucuucc
......................................................................aauugcacuuuucccgUccucu....................    21    1    Fm1
......................................................................aauugcacuuuucccggcUucu....................    5     1    Fm1
......................................................................aauugcacuuuucccUgccucu....................    1     1    Fm1
......................................................................aauugcacuuuucccgAccucu....................    19    1    Fm1
......................................................................aauugNacuuuucccggccucu....................    5     1    Fm1
......................................................................aGuugcacuuuucccggccucu....................    1     1    Fm1
......................................................................aauugcacuuuucccggccuAu....................    1     1    Fm1
......................................................................aauuAcacuuuucccggccucu....................    1     1    Fm1
......................................................................Nauugcacuuuucccggccucu....................    2     1    Fm1
......................................................................aauugcacuuuucccggccucG....................    116   1    Fm1
......................................................................aauugcUcuuuucccggccucu....................    2     1    Fm1
......................................................................aauugcNcuuuucccggccucu....................    1     1    Fm1
......................................................................aauugcacGuuucccggccucu....................    2     1    Fm1
......................................................................aauugcGcuuuucccggccucu....................    1     1    Fm1
......................................................................aauugcacuuuuAccggccucu....................    2     1    Fm1
......................................................................aauugcacuuuucccggccucC....................    12    1    Fm1
......................................................................aauugcacuuAucccggccucu....................    12    1    Fm1
......................................................................aauugcacuuuucAcggccucu....................    2     1    Fm1
......................................................................aUuugcacuuuucccggccucu....................    4     1    Fm1
......................................................................Uauugcacuuuucccggccucu....................    15    1    Fm1
......................................................................aauugcacuuuuccAggccucu....................    2     1    Fm1
......................................................................aauugcacuuCucccggccucu....................    9     1    Fm1
......................................................................aauugcaUuuuucccggccucu....................    2     1    Fm1
......................................................................Gauugcacuuuucccggccucu....................    2     1    Fm1
......................................................................aaAugcacuuuucccggccucu....................    2     1    Fm1
......................................................................aaCugcacuuuucccggccucu....................    1     1    Fm1
......................................................................aauugcacuuuucccAgccucu....................    1     1    Fm1
......................................................................aauugcacuuuuccUggccucu....................    3     1    Fm1
......................................................................aauugcacuuuucccggccucA....................    11    1    Fm1
......................................................................aauugcacuuGucccggccucu....................    12    1    Fm1
......................................................................aauugcacuuuucUcggccucu....................    4     1    Fm1
......................................................................Cauugcacuuuucccggccucu....................    1     1    Fm1
......................................................................aauAgcacuuuucccggccucu....................    2     1    Fm1
......................................................................aauugcacuuuucccggccucuC...................    7     1    Fm1
......................................................................aauugcacuuuucccggccucuU...................    247   1    Fm1
......................................................................aauugcacuuuucccggccucuA...................    31    1    Fm1
......................................................................aauugcacuuuucccggccucuAu..................    28    1    Fm1
......................................................................aauugcacuuuucccggccucuUu..................    126   1    Fm1
......................................................................aauugcacuuuucccggccucugu..................    1     0    Fm1
.......................................................................auugcacuuuucccggcUucu....................    1     1    Fm1
.......................................................................auugcacuuuCcccggccucu....................    1     1    Fm1
.......................................................................auugcacuuuucccggccuGu....................    1     1    Fm1
.......................................................................auugcacuuuucccggcGucu....................    1     1    Fm1
.......................................................................auugcacuuuucccggccucu....................    22    0    Fm1
.......................................................................auugcacuuuucccggccucug...................    1     0    Fm1
.......................................................................auugcacuuuucccggccucuA...................    2     1    Fm1
.......................................................................auugcacuuuucccggccucuU...................    1     1    Fm1
.......................................................................auugcacuuuucccggccucuAu..................    6     1    Fm1
.......................................................................auugcacuuuucccggccucugu..................    9     0    Fm1
.......................................................................auugcacuuuucccggccucugG..................    1     1    Fm1
........................................................................uugcacuuuucccggccucu....................    2     0    Fm1

.....................................................................gaauugcacuuuucccggccucu....................    1     0    Mm1
......................................................................aauugcacuCuucccggcc.......................    1     1    Mm1
......................................................................aauugcacuuuuccUggccu......................    1     1    Mm1
......................................................................aauugcacuuuucccggccu......................    296   0    Mm1
......................................................................aauugcacuuuucccgCccu......................    1     1    Mm1
......................................................................aauugcacuuuucccggAcu......................    2     1    Mm1
......................................................................Uauugcacuuuucccggccu......................    1     1    Mm1
......................................................................aauugcacuuuucccggccG......................    1     1    Mm1
......................................................................aauugcacuuAucccggccu......................    2     1    Mm1
......................................................................aUuugcacuuuucccggccu......................    1     1    Mm1
......................................................................aauugcacuuuucccgUccu......................    1     1    Mm1
......................................................................aauugcacuCuucccggccu......................    4     1    Mm1
......................................................................aauugcacuuuucccgCccuc.....................    1     1    Mm1
......................................................................aauugcacuuuucccggccuc.....................    2     0    Mm1
......................................................................aauugcacuuuucccggcUucu....................    1     1    Mm1
......................................................................aauugcacuuuucccggccucu....................    812   0    Mm1
......................................................................aauugUacuuuucccggccucu....................    2     1    Mm1
......................................................................aauugcacuuuucUcggccucu....................    1     1    Mm1
```

```
                                  Star                                 Mature
accccaaacccuggaucucccggcugcuuagcaaaggcugugauugguggcaaaauucuguugucaacaagaauugcacuuuucccggccucugugcuguggccaguucuucc
.......................................................................aauugcacuuuucccggGcucu....................  1     1    Mm1
.......................................................................Uauugcacuuuucccggccucu....................  3     1    Mm1
.......................................................................aaAugcacuuuucccggccucu....................  1     1    Mm1
.......................................................................aauugcacuuuucccgUccucu....................  2     1    Mm1
.......................................................................aauCgcacuuuucccggccucu....................  2     1    Mm1
.......................................................................aauugcacuuuucccggUcucu....................  5     1    Mm1
.......................................................................aauugcacuuuucccggccuGu....................  24    1    Mm1
.......................................................................aauugcacuuuucccgAccucu....................  4     1    Mm1
.......................................................................aauugcacuAuucccggccucu....................  1     1    Mm1
.......................................................................aauugcacuuuCcccggccucu....................  1     1    Mm1
.......................................................................aauugcacuuAucccggccucu....................  2     1    Mm1
.......................................................................aauugcacuuuucccggAcucu....................  3     1    Mm1
.......................................................................aauugcacuuuucccggccucA....................  4     1    Mm1
.......................................................................aauugcacuuGucccggccucu....................  1     1    Mm1
.......................................................................aauugcacuuCucccggccucu....................  1     1    Mm1
.......................................................................aauugcacuuuucccggccucuU...................  22    1    Mm1
.......................................................................aauugcacuuuucccggccucuA...................  10    1    Mm1
.......................................................................aauugcacuuuucccggccucuAu..................  1     1    Mm1
.......................................................................aauugcacuuuucccggccucuUu..................  8     1    Mm1
........................................................................auugcacuuuucccggccu......................  1     0    Mm1
........................................................................auugcacuuuucccggccucG....................  1     1    Mm1
........................................................................auugcacuuuucccggccuGu....................  1     1    Mm1
........................................................................auugcacuuuucccggccucu....................  8     0    Mm1

..................................aggcugugauugguggcaaaauucu......................................................  2     0    Mm2
......................................................................Caauugcacuuuucccggccu......................  4     1    Mm2
......................................................................gaauugcacuuuucccggccu......................  5     0    Mm2
......................................................................gaauugcacuuuucccggccucu....................  1     0    Mm2
......................................................................gaauugcacuuuucccggccucA....................  1     1    Mm2
......................................................................Caauugcacuuuucccggccucu....................  9     1    Mm2
.......................................................................aauugcacuuuucccggc........................  1     0    Mm2
.......................................................................aauugcacuuuucccggcc.......................  28    0    Mm2
.......................................................................aauugcacuuuuccUggccu......................  1     1    Mm2
.......................................................................aauugcacuuuAcccggccu......................  1     1    Mm2
.......................................................................aauugcacuuuucccggccu......................  3571  0    Mm2
.......................................................................aauugcacuuCucccggccu......................  7     1    Mm2
.......................................................................aauugcacuuAucccggccu......................  4     1    Mm2
.......................................................................aauGgcacuuuucccggccu......................  1     1    Mm2
.......................................................................aauugcacuuuucccgCccu......................  11    1    Mm2
.......................................................................aauugcCcuuuucccggccu......................  1     1    Mm2
.......................................................................aGuugcacuuuucccggccu......................  1     1    Mm2
.......................................................................aauugNacuuuucccggccu......................  2     1    Mm2
.......................................................................aauuCcacuuuucccggccu......................  1     1    Mm2
.......................................................................Gauugcacuuuucccggccu......................  1     1    Mm2
.......................................................................aauugcGcuuuucccggccu......................  1     1    Mm2
.......................................................................aauuUcacuuuucccggccu......................  3     1    Mm2
.......................................................................aauugUacuuuucccggccu......................  3     1    Mm2
.......................................................................aauugcacuuuucccggcUu......................  1     1    Mm2
.......................................................................aauugcaUuuuucccggccu......................  1     1    Mm2
.......................................................................aauugcacuCuucccggccu......................  3     1    Mm2
.......................................................................Uauugcacuuuucccggccu......................  4     1    Mm2
.......................................................................aCuugcacuuuucccggccu......................  1     1    Mm2
.......................................................................aauugcacuuuucccggccC......................  3     1    Mm2
.......................................................................aauugcacuuuCcccggccu......................  1     1    Mm2
.......................................................................aauugcUcuuuucccggccu......................  2     1    Mm2
.......................................................................aauugcacuuuucccggAcu......................  43    1    Mm2
.......................................................................aauugcacuuuucccggccG......................  104   1    Mm2
.......................................................................aauugcacuuuucccgAccu......................  8     1    Mm2
.......................................................................aauCgcacuuuucccggccu......................  1     1    Mm2
.......................................................................aauugcacuGuucccggccu......................  1     1    Mm2
.......................................................................aauugcacuuuucccggUcu......................  14    1    Mm2
.......................................................................aauugcacuuuucccgUccu......................  12    1    Mm2
.......................................................................aauugcacuuuucccggccA......................  16    1    Mm2
.......................................................................aauugcacuuuucccggGcu......................  12    1    Mm2
.......................................................................aauugcacCuuucccggccu......................  1     1    Mm2
.......................................................................aauAgcacuuuucccggccu......................  2     1    Mm2
.......................................................................aauugcacuuuuUccggccu......................  1     1    Mm2
.......................................................................aauugcacuuuucccggccCc.....................  1     1    Mm2
.......................................................................aauugcacuuuucccggccuU.....................  13    1    Mm2
.......................................................................aauugcacuuuucccgCccuc.....................  1     1    Mm2
```

Star Mature

```
accccaaacccuggaucucccggcugcuuagcaaaggcugugauuggugcaaaauucuguugucaacaagaauugcacuuuucccggccucugugcuguggccaguucuucc
......................................................................aauugcacuuuucccggccuc.....................    305    0    Mm2
......................................................................aauugcacuuuucccggccuA.....................    6      1    Mm2
......................................................................aauugcacuuuucccggAcuc.....................    4      1    Mm2
......................................................................aauugcacuuuucccggGcuc.....................    1      1    Mm2
......................................................................aauugcacuuGucccggccuc.....................    1      1    Mm2
......................................................................aauugcacuuuucccggccuG.....................    1      1    Mm2
......................................................................aauugcacuuuucccgAccuc.....................    1      1    Mm2
......................................................................aUuugcacuuuucccggccuc.....................    1      1    Mm2
......................................................................aauugcacuuuucccggUcuc.....................    4      1    Mm2
......................................................................aauugcacuuuuccAggccucu....................    3      1    Mm2
......................................................................Gauugcacuuuucccggccucu....................    5      1    Mm2
......................................................................aauuCcacuuuucccggccucu....................    5      1    Mm2
......................................................................aaAugcacuuuucccggccucu....................    5      1    Mm2
......................................................................aauCgcacuuuucccggccucu....................    5      1    Mm2
......................................................................aauugcacuuuucccgUccucu....................    72     1    Mm2
......................................................................aauugcacuuGucccggccucu....................    50     1    Mm2
......................................................................aauugcacuuuucccggccuAu....................    2      1    Mm2
......................................................................Cauugcacuuuucccggccucu....................    4      1    Mm2
......................................................................aauugcacuuAucccggccucu....................    65     1    Mm2
......................................................................aauugcGcuuuucccggccucu....................    6      1    Mm2
......................................................................aauugcacuuuucUcggccucu....................    12     1    Mm2
......................................................................aauugcacuuuucccggUcucu....................    166    1    Mm2
......................................................................aauugcacuuuucccggGcucu....................    118    1    Mm2
......................................................................aauugcacuuuuccUggccucu....................    15     1    Mm2
......................................................................aauugcacuuuAcccggccucu....................    3      1    Mm2
......................................................................aGuugcacuuuucccggccucu....................    8      1    Mm2
......................................................................Uauugcacuuuucccggccucu....................    37     1    Mm2
......................................................................aaGugcacuuuucccggccucu....................    7      1    Mm2
......................................................................aauugcacuuuuccGggccucu....................    3      1    Mm2
......................................................................aauugcaAuuuucccggccucu....................    3      1    Mm2
......................................................................aauuUcacuuuucccggccucu....................    8      1    Mm2
......................................................................aauAgcacuuuucccggccucu....................    3      1    Mm2
......................................................................aauugcacuuuucccggccuUu....................    12     1    Mm2
......................................................................aauugcacuuuucAcggccucu....................    10     1    Mm2
......................................................................aCuugcacuuuucccggccucu....................    4      1    Mm2
......................................................................aauugcacuuuuAccggccucu....................    2      1    Mm2
......................................................................aauugcacuAuucccggccucu....................    1      1    Mm2
......................................................................aauugcacuuuucccggccucu....................    32530  0    Mm2
......................................................................aauugcaGuuuucccggccucu....................    1      1    Mm2
......................................................................aauugcacuuuucccggccuGu....................    113    1    Mm2
......................................................................aauugcaUuuuucccggccucu....................    3      1    Mm2
......................................................................aauugcacuCuucccggccucu....................    6      1    Mm2
......................................................................aauugcacuuuucccUgccucu....................    3      1    Mm2
......................................................................aauugcacuuuucccggccucA....................    40     1    Mm2
......................................................................aauuAcacuuuucccggccucu....................    5      1    Mm2
......................................................................aauugcacuuuGcccggccucu....................    1      1    Mm2
......................................................................aauugcCcuuuucccggccucu....................    1      1    Mm2
......................................................................aauGgcacuuuucccggccucu....................    1      1    Mm2
......................................................................Nauugcacuuuucccggccucu....................    15     1    Mm2
......................................................................aauugcacuuuuUccggccucu....................    13     1    Mm2
......................................................................aaCugcacuuuucccggccucu....................    4      1    Mm2
......................................................................aauugcacuuuucGcggccucu....................    1      1    Mm2
......................................................................aauugcacuuuucccAgccucu....................    11     1    Mm2
......................................................................aauugcUcuuuucccggccucu....................    3      1    Mm2
......................................................................aauugcacuuuucccggcUucu....................    6      1    Mm2
......................................................................aauugUacuuuucccggccucu....................    12     1    Mm2
......................................................................aauugcacuuuucccgAccucu....................    87     1    Mm2
......................................................................aUuugcacuuuucccggccucu....................    6      1    Mm2
......................................................................aauugcacCuuucccggccucu....................    13     1    Mm2
......................................................................aauugcacGuuucccggccucu....................    1      1    Mm2
......................................................................aauugcacuuCucccggccucu....................    25     1    Mm2
......................................................................aauugcacuuuucccgCccucu....................    91     1    Mm2
......................................................................aauugcacuGuucccggccucu....................    4      1    Mm2
......................................................................aauugcacuuuucccggcGucu....................    1      1    Mm2
......................................................................aauugNacuuuucccggccucu....................    2      1    Mm2
......................................................................aauugcacuuuCcccggccucu....................    1      1    Mm2
......................................................................aauugGacuuuucccggccucu....................    1      1    Mm2
......................................................................aauugcacuuuucccggccCcu....................    2      1    Mm2
......................................................................aauugcacuuuucccggAcucu....................    288    1    Mm2
......................................................................aauugcacuuuucccggccucC....................    25     1    Mm2
```

```
                                  Star                                Mature
accccaaacccuggaucucccggcugcuuagcaaaggcugugauuggugcaaaauucuguugucaacaagaauugcacuuuucccggccucugugcuguggccaguucuucc
......................................................................aauugcNcuuuucccggccucu....................    4     1   Mm2
......................................................................aauugcacuuuucccggccucG....................    362   1   Mm2
......................................................................aauugcacuuuucccggccucuU...................    1178  1   Mm2
......................................................................aauugcacuuuucccggccucug...................    9     0   Mm2
......................................................................aauugcacuuuucccggccucuC...................    3     1   Mm2
......................................................................aauugcacuuuucccggccucuA...................    119   1   Mm2
......................................................................aauugcacuuuucccggccucuUu..................    343   1   Mm2
......................................................................aauugcacuuuucccggccucuAu..................    97    1   Mm2
......................................................................aauugcacuuuucccggccucugu..................    1     0   Mm2
......................................................................aauugcacuuuucccggccucuCu..................    2     1   Mm2
......................................................................aauugcacuuuucccggccucuUug.................    1     1   Mm2
.......................................................................auugcacuuuucccggccu......................    5     0   Mm2
.......................................................................auugcacuuuucccggccG......................    1     1   Mm2
.......................................................................auuCcacuuuucccggccuc.....................    1     1   Mm2
.......................................................................auugcacuuuucccggcGucu....................    1     1   Mm2
.......................................................................auugcacuuuucccggccuGu....................    5     1   Mm2
.......................................................................auugcacuuuucccggccucu....................    79    0   Mm2
.......................................................................auugcacuuuCcccggccucu....................    1     1   Mm2
.......................................................................auugcacuuuucccggcAucu....................    1     1   Mm2
.......................................................................auugcacuuuucccggccucuU...................    9     1   Mm2
.......................................................................auugcacuuuucccggccucuA...................    7     1   Mm2
.......................................................................auugcacuuuucccggccucug...................    4     0   Mm2
.......................................................................auugcacuuuucccggccucugu..................    5     0   Mm2
.......................................................................auugcacuuuucccggccucuUu..................    3     1   Mm2
.......................................................................auugcacuuuucccggccucuAu..................    3     1   Mm2
.......................................................................auugcacuuuucccggccucuguU.................    1     1   Mm2
........................................................................uugcacuuuucccggccucugu..................    2     0   Mm2
.........................................................................ugcacuuGucccggccucu....................    1     1   Mm2
..........................................................................gcacuuuucccggccucu....................    2     0   Mm2
```
